# The Role of Nutrition and Physical Activity in Modulating Disease Progression and Quality of Life in Multiple Sclerosis

**DOI:** 10.3390/nu17162713

**Published:** 2025-08-21

**Authors:** Cristina Grosu, Emilian Bogdan Ignat, Daniel Alexa, Alin Ciubotaru, Maria Magdalena Leon, Alexandra Maștaleru, Gabriela Popescu, Carmen Marinela Cumpăt, Laura-Elena Cucu, Mădălina Irina Smihor, Dan Trofin

**Affiliations:** 1 Department of Neurology, “Grigore T. Popa” University of Medicine and Pharmacy, 700115 Iasi, Romania; cristina.grosu@umfiasi.ro (C.G.); emilian.ignat@umfiasi.ro (E.B.I.); alexadaniel2004@yahoo.com (D.A.); alinciubotaru94@yahoo.com (A.C.); 2 Clinical Rehabilitation Hospital, 700661 Iasi, Romania; maria.leon@umfiasi.ro; 3 Department of Medical Specialties I, “Grigore T. Popa” University of Medicine and Pharmacy, 700115 Iasi, Romania; gabriela.popescu@umfiasi.ro; 4 Department of Medical Specialties III, “Grigore T. Popa” University of Medicine and Pharmacy, 700115 Iasi, Romania; marinela.cumpat@umfiasi.ro; 5 Faculty of Medicine, “Grigore T. Popa” University of Medicine and Pharmacy, 700115 Iasi, Romania; dudau.laura-elena@d.umfiasi.ro (L.-E.C.); mg-rom-31105@students.umfiasi.ro (M.I.S.); 6 Department of Biomedical Sciences, Faculty of Medical Bioengineering, “Grigore T. Popa” University of Medicine and Pharmacy, 700454 Iasi, Romania; trofin.dan@umfiasi.ro

**Keywords:** multiple sclerosis, diet, physical activity

## Abstract

Multiple sclerosis (MS) is a chronic, immune-mediated neurological disorder with increasing global prevalence. Emerging evidence underscores the role of lifestyle interventions (particularly diet and physical activity) in modulating disease progression and improving quality of life. This narrative review synthesizes current scientific literature on the effects of dietary interventions, including the Mediterranean, ketogenic, Swank, Wahls, gluten-free, and fasting-based diets, alongside various physical activity regimens. The Mediterranean and ketogenic diets show promise in reducing inflammation, enhancing neuroprotection, and improving metabolic health. Similarly, structured physical activity (including aerobic, resistance, sensorimotor, and mind–body exercises) demonstrates benefits in mobility, fatigue, and mental well-being. The review highlights the need for personalized, sustainable approaches that integrate nutritional and exercise-based strategies for optimal MS management in the long term.

## 1. Introduction

Multiple sclerosis (MS) is a demyelinating and neurodegenerative disease of the central nervous system, characterized by inflammation, immune dysregulation, and neuronal injury. While disease-modifying therapies remain the cornerstone of clinical management, sometimes they fall short in addressing the broader spectrum of symptoms and long-term disability progression. This has sparked growing interest in complementary lifestyle interventions, especially those involving diet and physical activity. Recent studies suggest that dietary patterns influence not only MS symptomatology but also underlying mechanisms such as inflammation, oxidative stress, mitochondrial dysfunction, and gut microbiota composition [1].

Similarly, structured physical activity has demonstrated the ability to enhance neuroplasticity, reduce fatigability, and improve both physical and mental health outcomes. Consequently, integrative care models that include lifestyle approaches are becoming increasingly significant in both clinical practice and research [2].

This narrative review aims to evaluate the current evidence surrounding dietary and physical activity interventions in MS. By analyzing multiple dietary approaches—including the Mediterranean, ketogenic, Swank, Wahls, gluten-free, and fasting-based diet regimens—and exercise modalities, we seek to provide practical and evidence-based recommendations to support MS patients in improving disease outcomes and quality of life.

## 2. Materials and Methods

**Figure 1:**
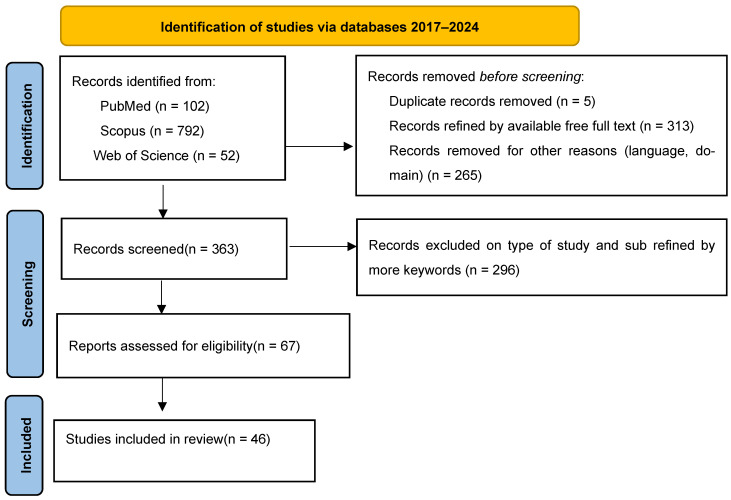
Flowchart of studies identification.

This narrative review is based on a comprehensive synthesis of peer-reviewed literature published between 2017 and 2024. Databases including PubMed, Scopus, and Web of Science were searched using combinations of keywords such as “multiple sclerosis,” “diet,” “Mediterranean diet,” “ketogenic diet,” “Swank diet,” “Wahls protocol,” “gluten-free,” “intermittent fasting,” “exercise,” and “physical activity.” (Figure 1).

Inclusion criteria comprised randomized controlled trials (RCTs), observational studies, systematic reviews, and meta-analyses that reported outcomes related to disease activity, inflammation, fatigue, neuroprotection, quality of life, or metabolic parameters in MS patients. Animal studies were included only when providing mechanistic insights relevant to human pathology. In our research, we included studies reporting positive, neutral, and negative outcomes, focused solely on pharmacological interventions without a lifestyle component in order to provide a balanced and comprehensive overview of the existing evidence.

The review was structured thematically, and data was analyzed in a descriptive approach to examine the benefits, mechanisms, and limitations of each dietary and exercise intervention. Recommendations were synthesized based on the strength of available evidence, clinical relevance, and feasibility of long-term adherence.

## 3. Benefits of the Mediterranean Diet in MS Patients

The Mediterranean diet (MD), already recognized for its cardiovascular and metabolic benefits, has been examined for its potential role in the management and prevention of MS. Characterized by a high intake of fruits, vegetables, legumes, whole grains, nuts, olive oil, and moderate consumption of fish and dairy, the MD provides a diverse array of nutrients and bioactive compounds that may confer neuroprotective, anti-inflammatory, and immunomodulatory effects. Although large-scale, long-term randomized clinical trials remain limited, a growing body of evidence supports the integration of MD principles into dietary recommendations for individuals living with MS [3].

### 3.1. Reduction in MS Risk, Disease Progression, and Disability

Epidemiological studies suggest that adherence to the MD may be associated with a lower risk of developing MS. In a recent investigation, Cavalla and Vercellino [4] found that individuals following a Mediterranean dietary pattern had a reduced likelihood of MS onset, independent of fish consumption. This finding implies that other dietary components (such as polyphenols, flavonoids, and antioxidants found in olive oil, citrus fruits, and grapes) may be responsible for the observed protective effect [4].

Dietary adherence to MD principles has also been associated with slower disease progression in MS. In a cross-sectional study by Felicetti et al. [5], patients with the RR (relapsing remitting) phenotype of MS who closely followed a Mediterranean-style diet exhibited lower scores on the Expanded Disability Status Scale (EDSS), indicating reduced physical disability. The intake of vegetables and fish appeared particularly beneficial [5]. These effects may be mediated by the MD’s ability to counteract oxidative stress and support mitochondrial function, both of which are critical in reducing neurodegenerative processes linked to MS [6].

### 3.2. Neuroprotection, Inflammation, and Gut Microbiota Modulation

The MD is rich in nutrients and compounds known to modulate inflammation and support neural function. Specifically, omega-3 fatty acids, polyphenols, and flavonoids (abundant in Mediterranean foods) are recognized for their anti-inflammatory and antioxidant properties. These compounds may help attenuate neuroinflammation, a central process in MS pathogenesis. Additionally, MD adherence has been linked to increased expression of brain-derived neurotrophic factor (BDNF), a key molecule in neuroplasticity and neuronal repair, which may contribute to improved cognitive function in MS patients [6].

The gut–brain axis is increasingly recognized as a relevant factor in MS, and the MD may contribute to improved neurological outcomes via modulation of the gut microbiome. The diet’s high content of dietary fiber and polyphenols promotes a diverse and anti-inflammatory microbial composition, which may help restore gut homeostasis and regulate immune function. These properties are particularly relevant in light of recent findings linking gut dysbiosis to MS pathogenesis and long-term evolution [7].

### 3.3. Quality of Life, Mental and Cardiometabolic Health Outcome

Beyond physical health, the MD appears to exert positive effects on mental health and quality of life in MS populations. Higher adherence to the MD has been associated with lower levels of depression and anxiety, as well as improved self-reported quality of life [8]. Moreover, diets rich in plant-based foods and omega-3 fatty acids (key features of the MD) have been correlated with reduced fatigue, a frequently reported and debilitating symptom among MS patients [5].

Obesity and cardiometabolic dysfunction are already known to exacerbate MS progression and symptom burden. The MD has been shown to promote weight stability, reduce insulin resistance, and improve lipid profiles, thereby contributing to lower systemic inflammation and reduced disease severity [8]. Furthermore, the MD supports blood pressure regulation and overall cardiovascular health, which is essential for managing common comorbidities in MS in the long term, as this is a chronic pathology [9].

In summary, while more robust clinical trials are needed to establish causal relationships and formalize clinical guidelines, current evidence supports the Mediterranean diet as a promising adjunctive intervention in MS management. Its multi-dimensional benefits (including inflammation reduction, neuroprotection, improved quality of life, and metabolic regulation) highlight the value of incorporating MD principles into dietary recommendations for individuals at risk of or living with MS.

## 4. Effects of the Ketogenic Diet in MS

The ketogenic diet (KD)—a high-fat, low-carbohydrate dietary regimen traditionally used in the management of epilepsy—has recently garnered attention for its potential therapeutic role in multiple sclerosis (MS). Preliminary studies suggest that the KD may exert neuroprotective, anti-inflammatory, and metabolic benefits, potentially contributing to improvements in neurological function, disease progression, and patient quality of life. However, despite encouraging findings, further long-term, large-scale studies are necessary to standardize protocols and validate clinical efficacy in broader MS populations [10].

### 4.1. Reduction in Inflammation and Neuroprotection

A growing body of research indicates that the KD modulates the inflammatory response implicated in MS pathology. Specifically, the diet has been shown to suppress the activation of microglia and astrocytes, both key mediators of neuroinflammation [11]. Additionally, KD enhances mitochondrial function and reduces oxidative stress, two critical contributors to neurodegeneration in MS [6]. In experimental models, KD administration led to reduced infiltration of immune cells into the central nervous system (CNS) and decreased myelin-reactive T cell responses, thereby attenuating demyelination and neuronal damage [12]. Furthermore, the diet has been linked to increased levels of brain-derived neurotrophic factor (BDNF), which supports neuronal survival and promotes neuroplasticity [6].

### 4.2. Improvements in Disability and Disease Progression

Clinical evidence also supports a potential role for KD in improving physical disability and altering disease trajectory in MS. Several studies have reported improvements in Expanded Disability Status Scale (EDSS) scores, suggesting enhanced mobility and decreased lesion formation among MS patients following a KD intervention [13]. In experimental autoimmune encephalomyelitis (EAE) models—a well-established animal model for MS—KD resulted in enhanced remyelination and a reduction in inflammatory cytokine expression, indicative of slowed disease progression [14]. Additionally, the diet has been found to modulate Tryptophan-Kynurenine metabolism, a pathway increasingly recognized for its role in immune regulation and neuroprotection in MS [6].

### 4.3. Impact on Fatigue and Quality of Life

Fatigue is a prevalent and debilitating symptom in MS, and KD has demonstrated promising results in this domain. The 6-month prospective study performed by Brenton et al. [13] found that a ketogenic diet was safe, well-tolerated, and had high adherence among 83 patients with RR MS. Participants experienced significant reductions in fatigue and depressive symptoms, accompanied by improvements in quality of life, neurological function, and inflammatory biomarkers [13]. The diet also improved exercise tolerance and decreased perceived stress, contributing to more favorable patient-reported outcomes [15]. These effects may, in part, be attributed to elevated levels of beta-hydroxybutyrate, a ketone body associated with enhanced cognitive function and neuroprotective effects [11].

### 4.4. Modulation of the Gut Microbiota and Metabolic Effects

Beyond its neurological benefits, KD may exert therapeutic effects through modulation of the gut microbiota, a critical factor in immune system regulation. Recent findings indicate that KD alters microbial composition in ways that reduce systemic inflammation and support immune balance in MS patients [16]. Additionally, KD is associated with improved insulin sensitivity and more favorable metabolic profiles, which may help to mitigate obesity-related inflammatory processes that can exacerbate MS symptoms [17]. By reducing oxidative stress and promoting mitochondrial biogenesis, the KD further enhances energy metabolism, contributing to overall improvements in cellular function and resilience [6].

### 4.5. Safety and Adherence Considerations

Short-term studies of the KD in MS populations have shown that the diet is generally well tolerated, with high adherence observed over a 6-month intervention period. Wetmore et al. [12] enrolled 65 RRMS patients into a 6-month prospective, intention-to-treat KD intervention. 21% of participants maintained strict adherence to the KD following the trial, while an additional 37% adopted a modified version of the diet, suggesting partial but sustained metabolic benefits [12]. Despite these encouraging adherence rates, the long-term safety and sustainability of the KD in MS remain uncertain, underscoring the need for further investigation into potential adverse effects, nutritional adequacy, and clinical outcomes over extended periods [6].

## 5. Effects of the Swank and Wahls Diets in MS

Two of the most studied dietary approaches in this context are the Swank diet and the Wahls diet, each of which offers a distinct nutritional framework with different therapeutic rationales and clinical outcomes.

The Swank diet, developed by Dr. Roy Swank, is characterized by a stringent limitation of saturated fat intake, based on early epidemiological observations suggesting a correlation between high saturated fat consumption and MS prevalence [18]. In a longitudinal clinical study, Swank reported that patients adhering to a diet restricted to ≤20 g of saturated fat per day experienced fewer relapses, slower progression of disability, and reduced mortality over a 50-year period [19]. More recent evidence from controlled trials supports the diet’s benefits, with participants demonstrating significant reductions in fatigue and improvements in quality of life (QoL) metrics when compared to baseline values [18].

Nutritionally, the Swank diet aligns in many respects with general dietary guidelines, emphasizing fruits, vegetables, and whole-grain intake. However, nutritional analysis suggests potential inadequacies, particularly in dietary fiber, potassium, and choline [19]. While it showed robust effects on fatigue, its impact on mental QoL was less substantial when compared to more nutrient-dense alternatives [18].

In contrast, the Wahls diet, developed by Dr. Terry Wahls, is a modified Paleolithic diet that excludes gluten, dairy, eggs, legumes, and processed foods while promoting a high intake of vegetables, fruits, and organ meats. The rationale for the Wahls diet includes the mitigation of inflammation, improvement of mitochondrial function, and modulation of the gut microbiota—all proposed mechanisms relevant to MS pathophysiology. Clinical investigations have shown that the Wahls diet leads to significant increases in serum levels of vitamins B12, D, and K1/K2, alongside reductions in inflammatory markers [20]. Furthermore, patients adhering to the Wahls diet report improvements in cognitive function, motor strength, and overall well-being [21].

Despite these promising outcomes, the restrictive nature of the Wahls diet raises concerns regarding the adequacy of certain micronutrients, particularly calcium and vitamin B12 due to the elimination of dairy and grains. This contrasts with the Swank diet, where vitamin A deficiency has been identified as a possible nutritional risk [20].

The Wahls versus Swank (WAVES) trial, a randomized parallel-arm controlled trial involving 77 individuals with RRMS phenotype, provided direct comparative data. Both dietary interventions resulted in significant reductions in fatigue and improvements in physical QoL at 12 weeks, with sustained benefits observed at 24 weeks. Notably, the Wahls diet demonstrated superior effects on mental QoL and yielded twice the improvement in physical QoL compared to the Swank diet [22]. However, neither diet significantly improved walking performance within the study period.

Psychological health outcomes have also been explored in the WAVES trial. Both diet groups exhibited reductions in depression and anxiety symptoms. Interestingly, these improvements were not correlated with changes in serum levels of vitamin B12, homocysteine, or folate, suggesting alternative mechanisms may underlie these effects [22].

In summary, both the Swank and Wahls diets have demonstrated potential to alleviate fatigue and enhance quality of life in individuals with MS. While each diet presents specific nutritional risks and benefits, the Wahls diet may offer broader impacts on mental health and inflammatory status. Nevertheless, individualized dietary planning and close nutritional monitoring remain essential and further large-scale, long-term randomized controlled trials are warranted to substantiate these findings and guide dietary recommendations in MS management.

## 6. Effects of a Gluten-Free Diet in MS

Recent research has begun to shed light on the potential role of dietary components, particularly wheat-derived proteins, in the progression and management of multiple sclerosis (MS). Among these, wheat amylase trypsin inhibitors (ATI)—non-gluten proteins found in wheat—have emerged as possible contributors to disease activity. In a mouse model of MS known as experimental autoimmune encephalitis (EAE), Zevallos et al. [23] demonstrated in an experimental study on EAE mice that dietary intake of ATI activates myeloid cells through toll-like receptor 4, thereby increasing inflammation in the central nervous system (CNS). Mice fed an ATI-containing diet experienced more severe disease progression compared to those consuming a gluten-only diet, reinforcing the concept that the gut–brain axis plays a significant role in inflammatory CNS diseases. These findings suggest that a diet excluding wheat and ATI could be beneficial for individuals with MS [23].

Despite these preclinical insights, the clinical evidence supporting the benefits of a gluten-free diet (GFD) in MS remains limited. A review of existing clinical data identified only a single non-randomized study that examined the effects of a GFD in MS patients. Although this study reported improvements in disability status and MRI findings, its methodological flaws limit the reliability of its conclusions [24]. Other dietary approaches, such as the Wahls Protocol—a modified paleolithic diet that excludes gluten—have shown promising results in terms of reducing self-reported fatigue, improving mood, and enhancing quality of life. However, the specific role of gluten elimination within these broader dietary frameworks remains unclear [24].

Adding further complexity, the relationship between MS, gluten sensitivity, and celiac disease (CD) has been the subject of numerous studies, yielding inconsistent results. While some research indicates slightly elevated levels of anti-gliadin antibodies (AGA) in MS patients, there is no strong evidence to support a significant association between MS and celiac disease. Moreover, large-scale population-based studies conducted in Denmark and Sweden found no increased prevalence of MS in individuals with celiac disease, nor vice versa [25].

Nonetheless, a few reports suggest a potential benefit of a GFD in MS. A systematic review identified two controlled trials and one cohort study that hinted at the favorable effects of gluten elimination on disease-related markers. In one small trial, MS patients adhering to a GFD exhibited lower disease activity on MRI scans and reduced disability scores compared to those following a regular diet. However, the lack of proper randomization in this study tempers the strength of its findings. Additional studies have proposed that gluten may contribute to MS pathogenesis by increasing blood–brain barrier permeability and stimulating autoreactive T cells, though these mechanisms remain hypothetical [25].

Given the current state of evidence, there is insufficient data to definitively recommend a GFD for non-celiac MS patients. Both Passali et al. [24] and Thomsen et al. [25] emphasize the urgent need for larger, well-controlled clinical trials to assess whether gluten elimination can meaningfully impact the course or symptoms of MS. Until such research is available, dietary recommendations for MS patients should be made with caution and tailored to individual needs and responses.

## 7. Effects of Fasting in MS

Recent studies have explored fasting-based dietary interventions—including intermittent fasting (IF) and fasting-mimicking diets (FMDs)—as potential therapeutic approaches for MS. IF regimens are generally categorized into 3 primary types: time-restricted eating (TRE), which involves consuming daily caloric intake within a consistent period, typically 8 h; alternate-day fasting (ADF), characterized by a rhythmic alternation between fasting and feasting days; and the 5:2 method, consisting of 5 days designated eating days interspersed with 2 non-consecutive fasting days each week.

These regimens, especially the TRE type, appear to influence key mechanisms relevant to MS pathophysiology, such as immune regulation, inflammation, demyelination, and neurodegeneration. Although further clinical validation is required, current evidence supports fasting as a promising adjunctive strategy for symptom management and disease modification in MS [26].

### 7.1. Reduction in Inflammation and Autoimmunity

One of the most supported effects of fasting in MS models is its anti-inflammatory and immunomodulatory properties. In the EAE mouse model of MS, intermittent caloric restriction through FMD (1/3 cal of control for 3 days, followed by ad libitum with normal chow for 4 days) resulted in significant reductions in disease severity, including decreased immune cell infiltration and reduced demyelination within the spinal cord. This was accompanied by a reversal in the accumulation of pro-inflammatory CD4^+^ T cells in the CNS, a hallmark of MS-related autoimmunity [27].

In human studies, which included mainly RRMS patients, IF has been shown to decrease levels of pro-inflammatory T cell subsets, particularly Th1 and Th17 cells, both of which are central to the autoimmune attack on myelin in MS. Additionally, these regimens appear to enhance immune tolerance, evidenced by an increase in naïve T-cell populations and a corresponding decrease in memory T cells—suggesting a shift toward a less autoreactive immune phenotype [28].

### 7.2. Promotion of Neuroprotection and Remyelination

Fasting also contributes to neuroprotective effects and remyelination, which are essential for slowing MS progression. In animal models, fasting has been linked to increased expression of brain-derived neurotrophic factor (BDNF), a key molecule in promoting neuronal survival and repair [27]. Moreover, the production of ketone bodies during fasting provides an alternative, efficient energy substrate for neurons and helps mitigate oxidative stress, a major contributor to neurodegeneration in MS [29].

Emerging evidence also suggests that fasting enhances the regeneration of oligodendrocyte precursor cells, a process essential for remyelination and restoration of axonal function in demyelinated lesions [30]. These findings highlight the potential of fasting to not only modulate immune responses but also promote structural repair within the CNS.

### 7.3. Improvement in Symptoms and Quality of Life

Clinical studies have begun to explore the effects of fasting on symptom burden and quality of life in individuals with MS. In one pilot interventional study on 12 RRMS patients, time-restricted eating (TRE)—a form of IF, restricting food intake to an 8 h window—was well tolerated (91%) and led to measurable improvements in fatigue and cognitive performance [31]. In addition, IF has been associated with improved mental well-being, including reductions in depression and anxiety, potentially through its effects on neurotransmitter regulation and reduced neuroinflammation [32].

Some MS patients have also reported improvements in mobility and reduced pain following IF interventions, suggesting broader effects on physical function and comfort [33]. These findings align with the observed reductions in inflammatory activity and neurotoxicity in response to fasting.

### 7.4. Modulation of Metabolism and Gut Microbiota

Fasting induces metabolic reprogramming that can attenuate risk factors associated with MS progression, such as insulin resistance and obesity-related inflammation [34]. IF has been shown to alter the composition of the gut microbiota, promoting the growth of beneficial bacterial species involved in immune regulation and barrier function. These microbiota changes may reduce intestinal permeability and systemic inflammation, both of which are implicated in MS pathogenesis [29].

Additionally, alterations in lipid metabolism during fasting correlate with reductions in oxidative stress and neuroinflammation, further supporting the neuroprotective profile of fasting-based interventions in MS [35].

### 7.5. Safety, Adherence, and Feasibility

The safety profile of fasting appears favorable in short-term interventions. A recent systematic review concluded that intermittent fasting is generally safe for MS patients, with no serious adverse events reported in clinical trials [33]. Time-restricted eating has shown particularly high adherence, making it a practical and sustainable option compared to more extreme or prolonged fasting protocols [35]. However, long-term adherence remains a challenge for some individuals, and certain patients may find strict fasting regimens difficult to maintain. Personalized, flexible dietary plans may therefore be necessary to support sustainable engagement with fasting approaches [34].

Below in Table 1, you can find a summary of the information described above.

In conclusion, fasting regimens, especially the TRE type in RRMS patients, demonstrate considerable potential in modulating immune activity, promoting neuroprotection, and improving patient-reported outcomes. Although findings from animal studies and preliminary clinical trials are encouraging, larger, long-term randomized controlled studies are warranted to determine optimal fasting protocols, assess long-term safety, and confirm therapeutic efficacy in diverse MS populations. In Table 2 below, we have summarized the key benefits, risks, and limitations of the main dietary interventions and their effects in MS patients.

## 8. Effects of Different Types of Exercises in MS

Physical activity has emerged as a vital component of comprehensive care in MS, offering multifaceted benefits across physical, cognitive, and psychological domains. While exercise does not directly alter the evolution of the disease in the short term, accumulating evidence underscores its capacity to modulate immune function, reduce inflammation, and improve quality of life. The following synthesis highlights the effects of various exercise modalities in MS management, drawing on current research.

### 8.1. Aerobic Exercise

Aerobic exercise, such as walking, cycling, and treadmill workouts, has demonstrated consistent benefits for individuals with MS. In a systematic review performed by Bellisario et al. [36] it is shown that regular aerobic activity reduces systemic inflammation, with trained individuals exhibiting lower levels of inflammatory markers compared to their sedentary counterparts [36]. Fatigue—a pervasive and disabling symptom in MS—is also substantially improved, with reductions of up to 30% reported in intervention trials [37].

In a recent meta-analysis by Gutierrez, meant to asses which type of physical exercise has the greatest positive effect on health-related quality of life (HRQoL) in people with MS, regardless of their phenotype, aerobic training was found to enhance cardiorespiratory fitness, cognitive performance, and mood, making it especially valuable for individuals with more severe disability, life SP phenotype. The conclusion was that sensorimotor training seems to be the most effective exercise to improve HTQoL for mild disease and aerobic and mind–body exercises to improve physical and mental HRQoL for severe disease [38]. While it does not appear to significantly alter MRI-based measures such as lesion load or brain volume in the short term, long-term functional gains have been observed [39].

### 8.2. Resistance Training

Resistance training, which includes mild weightlifting and bodyweight exercises, improves overall muscle strength, coordination, and balance in MS patients [40]. These improvements contribute to a reduction in spasticity and a lower risk of falls, particularly in individuals with moderate disability [41]. While not directly anti-inflammatory, resistance training supports functional independence and improves performance in daily tasks [39].

### 8.3. Sensorimotor Training

Sensorimotor training targets balance, proprioception, and neuromuscular control. It has shown the highest effect size among exercise interventions for improving overall quality of life in individuals with MS [38]. This modality is most effective in those with mild MS, as it enhances gait stability, coordination, and fall prevention. When combined with other forms of physical activity, it may also contribute to neuroplasticity and potentially slow disease progression [39].

### 8.4. Mind–Body Exercises: Yoga, Pilates, and Tai Chi

Mind–body exercises, such as yoga, Pilates, and Tai Chi, are particularly effective for improving mental well-being, stress regulation, and emotional balance. These forms of exercise have been associated with reductions in fatigue by up to 25%, and have shown benefits in cognitive function and emotional regulation [37,38]. They also improve muscle relaxation and flexibility, which may help alleviate spasms and stiffness [40].

### 8.5. Combined Aerobic and Resistance Training

Combined training protocols, integrating both aerobic and resistance components, are among the most effective strategies for improving both physical and mental outcomes in MS patients. This approach results in greater fatigue reduction than either modality alone, while also preserving endurance and cardiovascular health [37,41]. Combined training is especially recommended for individuals with moderate disability, as it enhances mobility, functional capacity, and independence in daily activities [39].

Chronic low-grade inflammation is a hallmark of MS, and regular exercise has been shown to modulate immune responses. Aerobic training, in particular, leads to decreased levels of pro-inflammatory cytokines such as IL-6, TNF-α, and IFN-γ. Simultaneously, both aerobic and resistance exercises stimulate the production of anti-inflammatory cytokines, including IL-10, which may have neuroprotective effects and contribute to symptom stabilization [36].

### 8.6. General Recommendations and Safety Considerations

Expert guidelines recommend that MS patients engage in at least 150 min of moderate-intensity exercise per week, combining aerobic, resistance, and flexibility training [41]. Exercise regimens should be personalized, considering symptom variability, fatigue levels, and mobility limitations [40]. For patients with significant mobility impairment, adaptive programs—such as aquatic therapy or seated strength training—can offer meaningful benefits while minimizing injury risk [41].

## 9. Key Contraindications and Cautions

While lifestyle interventions are generally safe in MS, specific diets and exercise regimens have important contraindications and precautions, or still have controversial aspects. Careful patient selection and monitoring are essential to avoid adverse outcomes [13,42]. Below we highlight key cautions for notable interventions:

### 9.1. Ketogenic Diet (KD)

Contraindicated in individuals with certain metabolic disorders or predispositions, KD’s high fat content and ketosis can exacerbate hyperlipidemia, hepatic steatosis, or gout, and it carries risk of metabolic acidosis and nephrolithiasis (kidney stones) [13]. Patients with a history of kidney stones or pancreatitis should generally avoid a KD, also pregnant or breastfeeding women, and typically underweight or frail patients (due to further weight loss risk). If pursued, close supervision by a dietitian and periodic monitoring of lipid profile, renal function, and micronutrient status are recommended. Common side effects include constipation, nausea, and the “keto flu” (transient fatigue, headache) during adaptation [42].

### 9.2. Fasting Protocols

IF or periodic fasting regimens are not appropriate for everyone. Absolute contraindications include pregnancy, type 1 diabetes, and active eating disorders (the caloric restriction can be dangerous in these contexts) [42]. Caution is warranted in patients on insulin or hypoglycemic medication (risk of hypoglycemia), and those with extreme fatigue or cognitive impairment, who may not safely adhere to fasting schedules. Ensure any patient attempting fasting is adequately hydrated to avoid hypotension and that they break fasts gradually to prevent electrolyte shifts. Minor adverse effects like headaches, irritability, or insomnia can occur during fasting periods [42]. Patients should be counseled to discontinue fasting during relapses or intercurrent illness (when nutritional support is needed for recovery).

### 9.3. Very Low-Fat Diets (e.g., Swank)

Extremely low-fat intake (<15–20 g saturated fat/day) can lead to deficiencies in essential fatty acids and fat-soluble vitamins (A, D, E, K) if not carefully managed. Clinicians should monitor for signs of malnutrition or vitamin deficiency in patients on long-term low-fat diets. Given that MS patients often have vitamin D insufficiency, a strict low-fat diet might require vitamin D and omega-3 supplementation to maintain adequate levels. Also, hypertriglyceridemia is a rebound effect that can occur in some individuals if the diet is high in refined carbs to compensate for low fat, but regular dietitian evaluation can help reduce these risks [19].

### 9.4. Wahls/Paleolithic Diet

The Wahls diet’s multiple eliminations (gluten-free, dairy-free, no processed foods) makes it highly restrictive, with adherence challenges and the potential for inadequate intake of certain nutrients (e.g., calcium if dairy is not consumed). Patients must ensure sufficient protein intake, because the diet is largely plant-based with some meats. Due to its complexity, the Wahls diet is not recommended for patients with limited social support or high stress, as the burden of food preparation is considerable. Any patient attempting it should ideally do so under nutritional supervision to prevent deficiencies (such as vitamin B12 or iodine if seafood is limited) [20].

### 9.5. Gluten-Free Diet (GFD)

GFD in individuals with multiple sclerosis (MS) who do not have celiac disease or confirmed gluten sensitivity may lead to unintended negative consequences. While a GFD is essential for managing celiac disease, in the context of MS, there is insufficient evidence to support its therapeutic use [25]. Gluten-free products are often lower in dietary fiber and may be higher in sugars and fats to improve palatability, potentially resulting in weight gain and metabolic disturbances [43]. This is particularly concerning for MS patients, as reduced fiber intake can negatively impact gut health and systemic inflammation, both of which are relevant to MS pathology [44]. Furthermore, restrictive diets such as a GFD may contribute to psychological distress, including anxiety around food choices and social isolation, particularly due to difficulties with dining out or participating in social events [45].

### 9.6. Intensive Exercise Regimens

High-intensity or high-frequency exercise programs should be introduced judiciously. Do not implement intense training during an acute MS relapse—during relapses, focus on rest and rehabilitation once the patient is clinically stable. Heat sensitivity (Uhthoff’s phenomenon) is a prime concern: strenuous exercise can raise core temperature and temporarily worsen neurological symptoms. This is not a true relapse but can be distressing; therefore, patients predisposed to heat intolerance should exercise in cool environments, use cooling vests or fans, and stay well-hydrated [40]. Another caution is overwork weakness—MS muscles can fatigue more quickly, and excessive exercise without adequate rest may lead to prolonged fatigue. To avoid injury, supervision by a physical therapist is recommended when initiating intensive regimens, especially resistance training with weights. Additionally, balance impairments necessitate caution with certain activities (for instance, running or cycling outdoors)—alternative modalities like stationary biking or pool exercises might be safer for some. Finally, any patient with significant cardiovascular comorbidity or deconditioning should have a cardiovascular screening before starting vigorous exercise, as MS patients may be less active for years and need gradual reconditioning.

## 10. Discussions

The analysis of dietary and physical activity interventions in MS yielded consistent evidence supporting their role in symptom management and quality-of-life improvement. The Mediterranean diet was associated with reduced inflammation, slower disability progression, and improved mental health outcomes [6]. The ketogenic diet demonstrated reductions in fatigue and depression, as well as enhanced remyelination and neuroprotection [13]. The Swank and Wahls diets were both effective in reducing fatigue, with the Wahls diet also improving mental quality of life and cognitive function [19]. Evidence supporting gluten-free diets was less consistent, although some small studies suggested potential benefits in select patients [25]. Fasting protocols, including intermittent and time-restricted eating, showed promise in reducing inflammatory markers and improving both mental and physical health outcomes [31].

Long-term feasibility and patient adherence to dietary interventions in MS vary considerably depending on the type of diet prescribed. The Mediterranean diet is generally regarded as the most sustainable, owing to its flexibility, palatability, and alignment with culturally familiar eating patterns. Its emphasis on whole foods, plant-based fats, and moderate protein intake makes it relatively easy to integrate into daily life, which likely contributes to higher adherence rates. In contrast, the ketogenic diet, while potentially beneficial for reducing neuroinflammation and improving fatigue in MS, poses significant challenges for long-term use due to its restrictive nature, high fat content, and potential side effects such as gastrointestinal discomfort and nutrient deficiencies. Similarly, intermittent fasting and caloric restriction regimens may show promise in modulating immune function and cellular repair processes, but their sustainability is questionable for many patients, especially those with coexisting fatigue, cognitive difficulties, or metabolic concerns.

The Wahls and Swank diets, both of which have gained popularity in the MS community, also present adherence challenges over time. The Wahls Protocol, which emphasizes high vegetable intake, elimination of processed foods, and often includes elements of paleo or ketogenic approaches, requires substantial lifestyle adjustments and food preparation, which may limit adherence in the long term. The Swank diet, characterized by very low saturated fat intake, is similarly demanding, and long-term commitment may wane due to its restrictive fat limitations and potential nutritional imbalances. While both diets have preliminary support for symptom management, robust longitudinal data on adherence and sustainability are limited. Overall, patient education, nutritional counseling, and individualized support are critical for improving long-term adherence and ensuring that dietary changes are both effective and maintainable in the context of MS management.

Regarding physical activity, aerobic and resistance training significantly improved fatigue, mobility, and overall muscle strength. Sensorimotor training was particularly effective for mild MS cases, enhancing balance and neuromuscular control [38]. Mind–body exercises such as yoga and tai chi improved emotional regulation and reduced spasticity [41]. Combined aerobic and resistance training provided the most comprehensive benefits across physical and psychological domains [37,39].

One particular situation that should be taken into consideration when giving recommendations about physical activity to MS patients is their level of disability. Edwards et al. [46] reviewed studies involving patients with significant mobility impairments, often with EDSS scores above 6.0. They found that even among individuals with severe disability, adaptive exercise modalities—such as body-weight-supported treadmill training and functional electrical stimulation cycling—led to meaningful improvements in physical function, fitness, and quality of life [46]. In their research, Baird et al. [47] emphasized the importance of recognizing heterogeneity in exercise responses among MS patients. Their narrative review argued that individual differences in disability level, symptom burden, and neurological impairment significantly influence the outcomes of physical activity interventions [47]. Halabchi et al. [40] also highlighted the efficacy of aerobic and resistance training in patients with lower disability scores, but pointed out the lack of robust data for higher-disability groups (EDSS > 7), stressing the need for more inclusive and stratified research [40]. These studies support the feasibility and potential benefits of structured exercise even in patients with limited mobility, although such programs often require specialized equipment and supervision.

An additional consideration related to physical exercise in individuals with MS is the risk of overtraining and possible worsening of symptoms in those with heat intolerance, where even a minimal increase in core body temperature (~0.2–0.5 °C) can lead to fatigue, balance impairment, blurred vision, and cognitive decline. This has led some clinicians and researchers to caution against overly intense or prolonged exercise, especially in warm environments. While the benefits of physical activity are well documented, there remains debate and controversy regarding how to balance intensity with the risk of provoking pseudo-relapses, particularly in heat-sensitive individuals. Silarova et al. [48], highlight that the severity and prevalence of exercise-induced heat sensitivity (EIHS) vary widely—from 29% to 80% of persons with MS—and depend on factors such as the type, duration, and format of exercise, as well as individual thermoregulatory capacity [48]. Cooling interventions, pre-cooling before activity, and monitoring intensity are all recommended to mitigate risks, yet standardized guidelines remain elusive, and empirical evidence is still limited regarding long-term safety across varying disability and heat tolerance levels.

Emerging evidence suggests that structured physical activity, particularly when combined with targeted nutritional support such as whey protein, essential amino acids (EAAs), or branched-chain amino acids (BCAAs), may offer synergistic benefits for individuals with MS. A recent case study by Ispoglou et al. [49] demonstrated that a 24-week program combining home-based exercise with EAA and vitamin D supplementation led to measurable improvements in lean muscle mass, strength, and functional capacity in a patient with MS [49]. Similarly, Zielińska et al. [50] reported cognitive benefits following dietary supplementation with tryptophan-enriched whey protein in MS patients, highlighting the broader therapeutic potential of high-quality protein sources [50]. While MS-specific trials that study nutritional supplementation and exercise remain limited, general findings from controlled studies in other populations consistently show that whey protein, due to its high leucine content and rapid absorption, stimulates muscle protein synthesis more effectively than other protein types. Collectively, these findings provide a rationale for integrating protein-based nutritional strategies into exercise interventions to counteract muscle loss and support physical function in people with MS.

The findings underscore the importance of integrating personalized nutrition and exercise regimens into MS care [51]. Diets rich in anti-inflammatory and neuroprotective compounds, such as the Mediterranean and ketogenic diets, demonstrated significant benefits across a range of clinical outcomes. However, each dietary approach has strengths and limitations. While the Wahls diet improved cognitive and mental health, its restrictiveness poses adherence challenges. Similarly, long-term sustainability of the ketogenic and fasting diets remains uncertain despite their short-term efficacy [52].

Exercise interventions, especially when tailored to individual capability and disease severity, consistently improved MS-related fatigue, mood, and functionality. Notably, combining aerobic and resistance training appears most effective. The emerging role of exercise in reducing inflammation adds further rationale for its inclusion in standard care [53].

## 11. Limitations

Limitations across reviewed studies include small sample sizes, short follow-up periods, and heterogeneity in intervention protocols. Long-term randomized controlled trials are essential to validate current findings and optimize individualized treatment strategies.

## 12. Conclusions

This review highlights the significant potential of dietary and physical activity interventions in managing multiple sclerosis. The Mediterranean and ketogenic diets, alongside structured exercise programs, offer clinically meaningful benefits in reducing inflammation, improving neurological function, and enhancing quality of life. Personalized approaches, accounting for individual preferences, symptom patterns, and metabolic profiles, are essential for long-term success. Future research should focus on conducting well-designed, large-scale randomized controlled trials to establish the efficacy and safety of specific dietary patterns and physical activity interventions in people with multiple sclerosis (MS). Comparative studies examining the long-term impact of diets such as the Mediterranean, ketogenic, Wahls, and Swank diets on disease progression, neuroinflammation, and quality of life are particularly needed. Additionally, research should explore the synergistic effects of combining diet and exercise, as well as the role of personalized interventions based on genetic, metabolic, and microbiome profiles. Standardized outcome measures and longer follow-up periods are essential to assess sustainability, adherence, and clinically meaningful benefits. Incorporating patient-reported outcomes and real-world data will also be critical to ensure that interventions are both effective and feasible in diverse MS populations. All this should be performed in order to establish standardized, evidence-based guidelines for integrating lifestyle modifications into MS management.

## Figures and Tables

**Table 1:** Studies that evaluate the fasting regimen in MS patients.

Study (Year)	Study Type	Duration	Fasting Regimen	MS Patients (*n*)	MS Type	Adherence
Morales-Suarez-Varela et al., 2021 [26]	Systematic Review of RCTs	Varied	Intermittent fasting	Not specified	Multiple (not detailed)	Varied; not uniformly reported
Fitzgerald et al., 2022 [28]	Randomized Controlled Trial (RCT)	12 weeks	Intermittent Calorie Restriction	36	Relapsing-remitting MS (RRMS)	High (monitored and reported)
Wingo et al., 2023 [31]	Feasibility Study	8 weeks	Time-Restricted Eating	19	Not specified	Good (based on reported compliance)
Lin et al., 2024 [33]	Systematic review	Varied	Intermittent Fasting	Included studies (n not stated)	Primarily RRMS	Generally high in included studies
Fitzgerald et al., 2018 [34]	Randomized Controlled Trial	8 weeks	Intermittent vs. Daily Calorie Restriction	36	Relapsing-remitting MS (RRMS)	Good (monitored and analyzed)
Roman et al., 2020 [35]	Feasibility Study	Short-term (5–7 days cycles)	Fasting-Mimicking Diets	16	Relapsing-remitting MS (RRMS)	Acceptable; tolerability reported

**Table 2:** Summary of Dietary Interventions and Their Reported Effects in MS Patients.

Diet Type	Type of Studies	Types of MS Form	Key Benefits	Risks/Limitations	References
Mediterranean	Observational, cross-section, narrative reviews	RRMS SPMS	↓ Inflammation, ↑ BDNF, ↓ Disability (EDSS), ↑ QoL	Limited RCTs; adherence varies	Di Majo et al., 2022 [6]; Felicetti et al., 2022 [5]; Dakanalis et al., 2024 [8]
Ketogenic	Phase II clinical trial, narrative review, survey	RRMS	↓ Inflammation, ↑ Mitochondrial function, ↓ Fatigue, ↑ Neuroprotection	GI issues, hard adherence, potential nutrient deficits	Brenton et al., 2022 [13]; Di Majo et al., 2022 [6]; Wetmore et al., 2023 [12]
Wahls Diet	RCT, nutritional intake analysis	RRMS	↓ Fatigue, ↑ Mental health, ↑ Nutrient density	Highly restrictive, risk of calcium/B12 deficiency	Wahls et al., 2021 [18]; Titcomb et al., 2021 [20]
Swank Diet	Descriptive diet comparison, RCT	RRMS	↓ Fatigue, slower disability progression	Risk of EFA & vitamin D deficiency	Chenard et al., 2019 [19]; Wahls et al., 2021 [18]
Gluten-Free	Review	RRMS	Possible ↓ Inflammation; ATI removal may help	Low fiber, ↑ weight gain risk, no strong evidence in non-celiac MS	Thomsen et al., 2019 [25]; Passali et al., 2020 [24]
Fasting	RCT, feasibility study	RRMS	↓ Inflammation (Th1, Th17), ↑ BDNF, ↓ Fatigue	Hard to sustain, not for Type 1 diabetes, eating disorders	Bai et al., 2021 [27]; Fitzgerald et al., 2022 [28]; Wingo et al., 2023 [31]

## Data Availability

Not applicable.
